# Renal histological findings in a patient with acute renal injury associated with purpura fulminans: a case report

**DOI:** 10.1590/2175-8239-JBN-2018-0074

**Published:** 2018-09-13

**Authors:** Julia Izadora da Silva Martins, Isabela Maria Bertoglio, Amanda Carolina Damasceno Zanuto Guerra, Mariana Espiga Maioli, Vinicius Daher Alvares Delfino

**Affiliations:** 1 Universidade Estadual de Londrina Programa de Clínica Médica LondrinaPR Brasil Universidade Estadual de Londrina, Programa de Clínica Médica, Londrina, PR, Brasil.; 2 Universidade Estadual de Londrina LondrinaPR Brasil Universidade Estadual de Londrina, Londrina, PR, Brasil.

**Keywords:** Purpura Fulminans, Thrombotic Microangiopathies, Acute Kidney Injury, Biopsy

## Abstract

**Introduction:**

Purpura fulminans (PF) is a rapid progressive thrombotic disease in which hemorrhagic infarction of the skin and disseminated intravascular coagulation (DIC) occurs. It can potentially cause acute kidney injury (AKI). However, there is no description in the medical literature of renal histological findings of PF.

**Case report:**

A 20-year-old female patient, previously healthy, was admitted to the emergency department (ED) with odynophagia, fever, generalized myalgia and anuria, which evolved with the appearance of purpuric plaques on the face and limbs. She required dialysis on admission. Laboratorial tests showed anemia, leukocytosis, thrombocytopenia, and elevation of lactic dehydrogenase (LDH). The purpuric lesions became bullous with ruptures and then necrotic and erosive, reaching the dermis, subcutaneous tissue and musculature, until bone exposure. There was no improvement with initial antibiotic therapy aimed at the treatment of meningococcemia. Thrombotic microangiopathy (TMA) and PF were then suspected. The patient remained in daily dialysis, requiring plasmapheresis. After sustained improvement of the thrombocytopenia, she underwent renal biopsy, which was not compatible with TMA, characterizing possible PF. A complete recovery of the renal function was achieved and cutaneous sequels were treated with grafts.

**Conclusion:**

When thrombotic and hemorrhagic phenomena overlap, obtaining a renal biopsy can be difficult. However, in the presented case, the biopsy allowed the exclusion of AKI caused by TMA, presenting for the first time, histological findings compatible with PF.

## INTRODUCTION

Purpura fulminans (PF) is a rapid progressive thrombotic disease in which hemorrhagic infarction of the skin and disseminated intravascular coagulation (DIC) occurs. The condition can also evolve into multiple organ failure or venous thrombosis of large vessels.^1^ PF lesions can be clinically distinguished from simple skin hemorrhage for usually being well demarcated, hardened, and with an erythematous circumferential area. With time, the lesions interconnect and evolve into tissue necrosis.^2^

There are three categories of PF: neonatal PF, associated with hereditary deficiency of anticoagulants; acute infectious PF or sepsis-associated PF, which causes DIC; and, idiopathic PF, subdivided into post-infectious PF (commonly associated with *Varicella* and *Streptococcus* infections) and PF of unknown etiology.^1^^-^5 Acute infectious PF is considered a synonymous for severe meningococcemia, since 10-20% of acute meningococcemia cases result in PF. However, there are records of bacteremia by *Staphylococcus aureus* and *Streptococcus pneumoniae* with PF as a complication;^6^^,^^7^ PF was also reported in some viral infections, such as dengue.^8^

Thrombotic microangiopathy (TMA) usually results from microangiopathic hemolytic anemia (MAHA), thrombocytopenia, and the presence of schistocytes in peripheral blood. Histologically, it is characterized by damaged and edematous endothelial cells, without arterioles and capillaries, and clusters of platelets and hyaline thrombi that cause partial or complete microvascular occlusions.^9^ In the kidneys, edematous endocapillary cells (endotheliosis), fibrin thrombi, platelet clusters, fibrosis of the intima and a membranoproliferative pattern can be found. The diseases associated with TMA include thrombotic thrombocytopenic purpura (TTP), hemolytic uremic syndrome (HUS), malignant hypertension, antiphospholipid syndrome, preeclampsia/HELLP syndrome, HIV infection, and others.^9^ Regardless of the etiology, TMA is a hematological emergency that requires immediate treatment,^10^ and it can be classified into primary and secondary TMA. Primary TMA occurs spontaneously, without an associated cause; the secondary form occurs in gestation, autoimmune diseases, use of certain medications and malignant disease, as examples.^11^

HUS and TTP are the prototypes of MAHA.^9^ HUS is characterized by MAHA, thrombocytopenia and renal dysfunction. In most cases, the etiologic agent is *E. coli* producing Shiga toxin. However, in a minority of cases, atypical HUS (aHUS) may occur - a rare genetic disorder characterized by complement-mediated TMA resulting from mutations affecting the regulation of the alternative complement pathway.^12^ In TTP, hemolytic anemia and thrombocytopenia can be found and fever may occur; renal impairment and neurological manifestations are variable. TTP is associated with deficiency or dysfunction of the ADAMTS13. TTP may be congenital (mutations in ADAMTS13) or acquired (autoantibodies). Like the aHUS, infections and other stressors can trigger TTP, but in aHUS the ADAMTS13 levels are normal.^13^ PF can also trigger hemolytic anemia or other forms of anemia.^14^^,^^15^ leading to the combination of tissue extravasation, external losses, and microangiopathic hemolysis, which are the characteristics of the disease.^15^

All these alterations are potentially the cause of acute kidney injury (AKI); however, in view of a clinical situation in which the thromboembolic and hemorrhagic risks are present, it may be difficult to obtain renal biopsy to define the cause of injury. There is no description in the reviewed medical literature of the renal histological findings of PF.

## CASE REPORT

**Figure 1:**
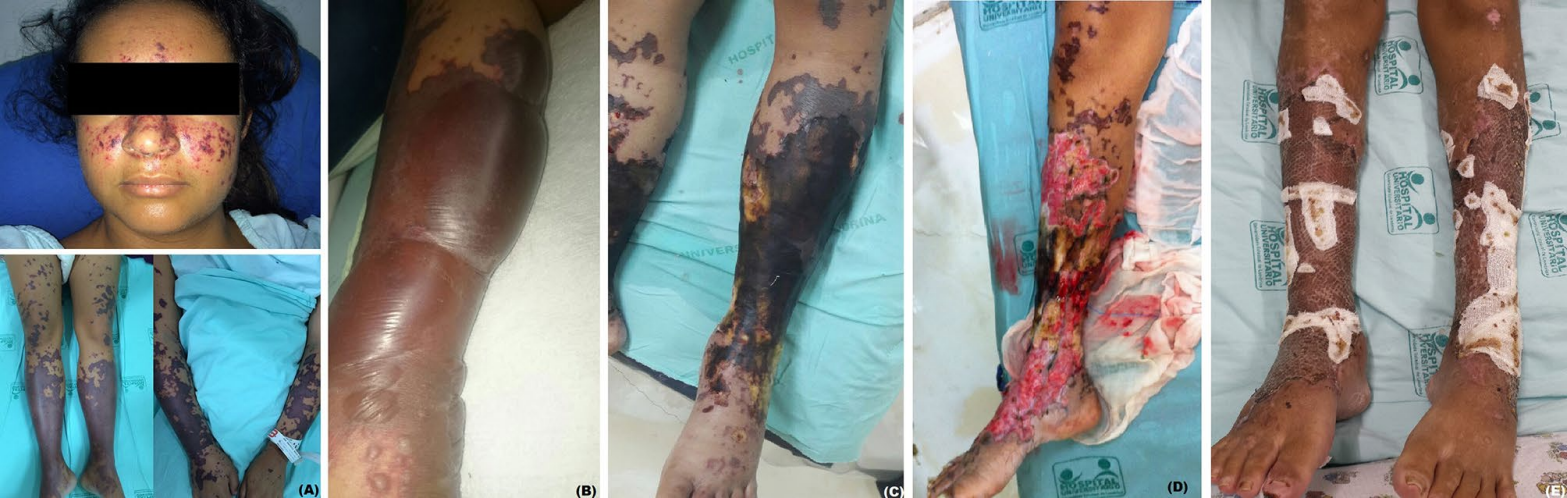
Evolution of skin lesions: petechiae and purpuric plaques (A), blisters (B), necrosis (C), bone exposure (D), and skin grafting results (E).

A 20-year-old black woman, previously healthy, was admitted to the emergency department (ED) of the University Hospital of Londrina (UH) complaining of odynophagia, a single episode of unmeasured fever and generalized myalgia with onset five days past. She was self-medicated with dipyrone and ibuprofen but did not get better and sought a basic health unit where amoxicillin was prescribed. On the same day, she reported appearance of petechiae on the face, and upper and lower limbs. She sought the secondary hospital, where she developed an large amount of petechiae (Figure 1), followed by confluence to clusters, worsening of general condition, and alteration of laboratory tests results; she was then referred to the UH. In the physical examination, she was slightly hypertensive (150/70 mmHg), pale, tachycardic, hyperemic, with purulent tonsils, cyanosis of the extremities, and purpuric plaques on the face and upper and lower limbs. The patient was anuric and required dialysis on admission. Laboratory tests showed anemia (Hb 10.7 g/dL), leukocytosis (44,990/µL), thrombocytopenia (24,000/µL), impaired renal function (Cr 4.15 mg/dL), hyponatremia and hyperkalemia, and elevation of LDH to 3,579 U/L.

The initial diagnostic hypotheses on admission were meningococcemia and staphylococci. Blood cultures were collected and antibiotic therapy (ceftriaxone and vancomycin) was started. Despite the antibiotics, the patient continued with worsening of the clinical condition, hypotension, tachycardia, and tachypnea, and purpuric lesions became bullous (Figure 1). The presence of schistocytes and elevated serum LDH levels indicated a possible TMA. The patient did not present neurological alterations, thus excluding the diagnosis of thrombotic TTP. Considering the hypothesis of aHUS, we started investigating the possible causes of TMA. All blood cultures and rheumatologic tests were negative. The serology performed was also negative, except IgM positive for dengue, collected on the sixth, twelve and twenty-eight days of medical history, all positive. The result for arboviruses (dengue 1, 2, 3 and 4, chikungunya and zika) was negative. However, this was collected on the sixth day of the onset of the disease, and ideally, the arbovirus survey sample is collected on the first day of symptoms and is acceptable up to the fifth day of the disease.

**Figure 2:**
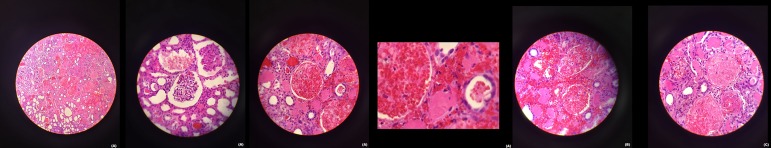
Renal Biopsy: extensive areas of ischemic and hemorrhagic infarction (A), interstitial hemorrhage (B), and fibrin thrombi in medium-sized vessels (C).

The patient completed the antibiotic regimen and afterwards, she was maintained only in supportive treatment, performing daily hemodialysis due to anuria and frequent need for transfusion of red blood cells due to anemia. On the fifteenth day of hospitalization, after sustained improvement in thrombocytopenia, she underwent renal laparoscopic biopsy, which identified extensive areas of ischemic and hemorrhagic infarction, interstitial hemorrhage, and medium-sized vessels with fibrin thrombi (Figure 2). Skin lesions were also analyzed by biopsy, which identified inflammatory infiltrate with extensive necrosis in adipose tissue, absence of signs of vasculitis in muscles, and absence of dermis and local epidermis surrounded by areas of epidermal repair. These lesions evolved with rupture of blisters and necrotic areas that progressively deepened, reaching the dermis, subcutaneous tissue, musculature, and tibial bone exposure (Figure 1). There was a need for frequent debridement of the necrotic areas in lower limbs, performed by the plastic surgery team of the burn treatment center of the service. As there was incessant progression of the lesions in legs and feet soft tissues, it was considered that the underlying disease that was causing the necrosis was be active. All available markers were negative for vasculitis and there was no purulent secretion or positive culture that would justify the clinical evolution. On the 32^nd^ day of hospitalization, plasmapheresis was chosen as an alternative measure since there was association with TMA, and initially, aHUS was one of the diagnostic hypotheses. Around the 40^th^ day of hospitalization, the patient started to show a significant diuresis and the necrotic areas in the legs stabilized. Hemodialysis was suspended and the plastic surgery team started skin grafting with good response (Figure 1). The patient was discharged after 68 days of hospitalization and the diagnosis of TMA and idiopathic PF associated with AKI was established.

## CONCLUSION

This study presents a report of a patient with severe acute onset and rapid evolution of purpuric lesions complicated by AKI requiring hemodialysis. Although PF is considered a thrombotic disease, with indication of full anticoagulation therapy, in this case, the use anticoagulant was not possible due to its association with hemolytic and thrombocytopenic conditions, making management more difficult. Initially, it was believed to be a self-limiting disease, since the investigation of infectious causes was negative, including a false positive test for dengue. However, the active disease, with risk of lower limb amputation due to progressive necrosis in bone, caused concern. Plasmapheresis was indicated as an alternative measure, in order to stop the aggressive progression of the cutaneous disease. In addition, the association with TMA and the hypothesis of aHUS that had been initially considered corroborated this indication. There was a clinical response to treatment, stabilizing the areas of necrosis, allowing the skin grafting. The renal biopsy, which is very difficult to obtain in these cases, was important and allowed the differentiation of the possible causes of AKI, with histological findings compatible with TMA, such as microthrombus in the lumen of the glomerular capillaries, arterioles and arteries, myointimal proliferation, leading to glomerular ischemia and tuft retraction.^16^ The presence of these hemorrhagic findings in the interstitium led us to consider, therefore, the diagnosis of AKI caused by PF. Further studies are needed to corroborate these findings, although obtaining a renal biopsy in these cases is still of great technical difficulty.
